# Female reproductive competition explains variation in prenatal investment in wild banded mongooses

**DOI:** 10.1038/srep20013

**Published:** 2016-01-28

**Authors:** Emma L. Inzani, Harry H. Marshall, Jennifer L. Sanderson, Hazel J. Nichols, Faye J. Thompson, Gladys Kalema-Zikusoka, Sarah J. Hodge, Michael A. Cant, Emma I. K. Vitikainen

**Affiliations:** 1 Centre for Ecology and Conservation, University of Exeter, Penryn Campus, Cornwall TR10 8FE

## Abstract

Female intrasexual competition is intense in cooperatively breeding species where offspring compete locally for resources and helpers. In mammals, females have been proposed to adjust prenatal investment according to the intensity of competition in the postnatal environment (a form of ‘predictive adaptive response’; PAR). We carried out a test of this hypothesis using ultrasound scanning of wild female banded mongooses in Uganda. In this species multiple females give birth together to a communal litter, and all females breed regularly from one year old. Total prenatal investment (size times the number of fetuses) increased with the number of potential female breeders in the group. This relationship was driven by fetus size rather than number. The response to competition was particularly strong in low weight females and when ecological conditions were poor. Increased prenatal investment did not trade off against maternal survival. In fact we found the opposite relationship: females with greater levels of prenatal investment had elevated postnatal maternal survival. Our results support the hypothesis that mammalian prenatal development is responsive to the intensity of postnatal competition. Understanding whether these responses are adaptive requires information on the long-term consequences of prenatal investment for offspring fitness.

Intrasexual competition is usually most severe among males, because males generally have higher variance in reproductive success than females^1^. This is manifested through conspicuous traits such as aggression and weaponry^2^. In cooperatively breeding species, female competition for reproduction is also intense, leading to overt and sometimes aggressive competition^3^. Because the cost of producing young is higher for females compared to males, theory suggests females will often resolve conflict without recourse to overt violence, for example, through the use of signals or threats^4^.

Recently, it has been suggested that females may compete over reproduction via maternal effects on offspring growth. In hyenas (*Crocuta crocuta*) and red squirrels (*Tamiasciurus hudsonicus*), for example, there is evidence that mothers prime their offspring to face competitive social environments through hormonal signaling (androgens or glucocorticoids[GCs]^5^,^6^). Experimental manipulations of population density in other taxa have also shown that offspring size is increased in response to adverse conditions (increased competition)^7^,^8^,^9^,^10^,^11^. These effects can be interpreted as a form of ‘predictive adaptive response’ (PAR), whereby mothers (or, potentially, offspring themselves) are hypothesized to adjust the developmental trajectory to ensure a match between offspring phenotype and the environment experienced postnatally or in later life^12^,^13^,^14^,^15^. However, no study of wild mammals has directly tested whether mothers adjust prenatal investment according to the postnatal environment, and in particular the intensity of reproductive competition.

We carried out this test in a wild cooperatively breeding mammal, the banded mongoose (*Mungos mungo*)^16^. Banded mongooses are small diurnal carnivores which live in stable groups of ~20 adults plus pups. Multiple females (mean = 3.5 females, range 1 to 13) give birth together in each breeding attempt, usually on the same day. Groups breed on average four times per year, experiencing considerable variation in environmental conditions (i.e. rainfall) which is strongly linked to invertebrate prey abundance^17^,^18^. Females compete postnatally using infanticide, but can escape infanticide through birth synchrony^19^. Offspring compete for access to lactating females and helpers (called “escorts”) who provision and protect pups after they emerge from the den. There is also evidence of prenatal maternal impacts on offspring competitiveness: mothers that are heavier at conception produce larger pups which have competitive advantage when competing for alloparental care; increasing pup survival^20^.

We carried out 360 ultrasound scans on 59 breeding females from 8 groups of banded mongooses to test (1) whether mothers adjust prenatal investment in response to reproductive competition, and (2) the consequences of variation in prenatal investment for mothers and offspring.

## Methods

### Study site

We studied a population of banded mongooses living on and around Mweya Peninsula, Queen Elizabeth National Park (QENP), Uganda (0°12′S, 27°54′E) between May 2000 and November 2013. For a detailed description of the climate, habitat and the population see Cant *et al.* 2013^18^. Rainfall data was provided by Uganda Institute of Ecology Meteorological Station and, later, using a rain gauge.

### Study population

All individuals in the population are known and individually marked with either colour-coded collars (7 g) or unique shave patterns (for details of trapping protocol and anesthesia are given elsewhere; Ketamine^21^; Isoflurane^22^). The identity of breeding females was determined from changes in body shape, ultrasound scans and palpation^23^,^24^. Each group was visited daily to determine accurate parturition dates. Since parturition can be determined precisely but conception cannot, we calculated the age of fetuses retrospectively assuming an average 60 day gestation (the mean period between peak mate guarding and birth^23^). Group size and the number of females were counted as the total number of individuals or females over 1 year old in each group for each communal litter. Individuals are habituated to step onto electronic scales to determine an accurate weight which allows regular weighing events without capture. Female weight at the time of conception was calculated using the closest weighing event prior (±10 days from conception) to the estimated conception date; if possible weights for all females within the same group came from the same weighing event.

### Measuring fetus size and number

Number of fetuses was counted under anesthesia by palpitating the abdomen, and a cross-sectional ultrasound scan of each fetus was obtained using an ultrasound scanner (SIUI CTS-900V, UK) and ultrasound gel (Anagel, UK). Trapping females within the last few weeks of pregnancy was avoided and most trapping was conducted 3-4 weeks after oestrus. Previous study has shown no adverse effects of trapping and palpitating pregnant females^24^. The age of the fetus at the time of the ultrasound scan was calculated retrospectively from the litter birth date and the scan date, assuming a gestation length of 60 days (average female gestation length^23^).

**Figure 1:**
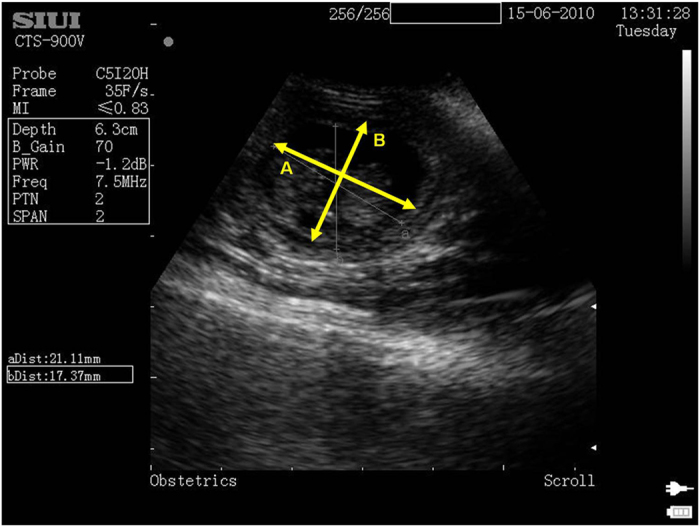
Cross-sectional ultrasound scan of individual fetus with 2 perpendicular measurements A and B used to calculate the cross-sectional area (A/2 × B/2 × π).

We used the cross-sectional area (mm^2^) of each fetus as measured from the ultrasound images as an estimate of fetus size. Fetuses were measured on average at 30 ± 7 (mean ± sd) days post conception when they are still roughly spherical in shape to minimize noise arising from different angles of the scan cross-section. The outline of a fetus was identified by the black pixilation of the fluid-filled amniotic sac and the white pixilation of the womb tissue and the amniotic sac membrane around the fetus. The mean of two perpendicular measurements of the diameter were taken using the computer software Image J (1.47c^25^) and used to calculate the elliptical area of the fetus (see Fig. 1).

### Statistics

We analyzed fetus sizes and the number of fetuses using general linear mixed models (LMMs) and generalized linear mixed models (GLMMs) in R version 3.1.0 using lme4 package R1.1-6^26^,^27^. GLMMs had either a poisson error structure with log-link function or binomial error structure with logit link function. Female, litter and group identities were included as random factors in analyses to account for the repeated sampling. Fixed terms included were female weight at conception, female age (months), number of adult females present in the group, group size and the total rainfall during gestation (ml). Because groups were trapped at different stages of pregnancy, fetus age (days) was included as a covariate when analyzing fetus size. Correlations between variables fitted in the same models as fixed effects were lower than the levels indicated by Freckleton^28^ to cause model fitting issues such as variance inflation in effect estimates (max *r* = 0.48). We obtained a minimal model via sequential removal of least significant factors, starting with 2-way interactions. Each factor was then added back into the minimum model in order to confirm removal was not contingent on the order of removal^29^.

To investigate if mothers adjust their prenatal investment in response to reproductive competition we estimated total prenatal investment by multiplying the average fetus size by the number of fetuses carried for each pregnancy. Variation in prenatal investment could be due to individual female adjustment in response to competition (a within-individual effect) or be the result of consistent differences between individuals. We tested the relative importance of within- versus between-individual effects using the method described by van de Pol & Wright^30^, which separates out the effect sizes in the fitted model attributable to variation within versus between individuals. To test the consequences of variation in prenatal investment for mothers and offspring we focused on pup survival to 3 months (y/n) using logistic regression, and pup weight (controlled age at capture <90 days) as well as female reproductive effort and survival. Maternity assignments for pups were based on 43 microsatellite loci as described in Sanderson *et al.*^31^. As individual fetus scans cannot be matched to pups an average fetus size was used in these analyses. Relative fetus size was calculated as the average fetus size in each female’s litter relative to average fetus size for all females within a breeding attempt. We tested whether prenatal investment predicted female participation in the next group litter (y/n) using a GLMM with binomial errors. We tested whether there was a trade-off between current investment in reproduction and female survival using Cox regression with backward selection of terms (Wald Chi-square). This analysis included total group size, number of females, and the average fetus size and number of fetuses as predictors, and to avoid repeat sampling used only the last reproductive event on record for each female. This analysis was conducted in SPSS 21.0.0.0^32^.

### Ethical Statement

Research was carried out under a permit from Uganda Wildlife Authority (UWA) and Uganda National Council for Science and Technology (UNCST), and all methods approved by UWA, UNCST and the Ethical Review panel of the University of Exeter. All methods were carried out in accordance with the Guidelines for the Treatment of Animals in Behavioural Research and Teaching published by the Association for the Study of Animal Behaviour^33^.

## Results

### (1) Do mothers adjust prenatal investment in response to reproductive competition?

**Figure 2:**
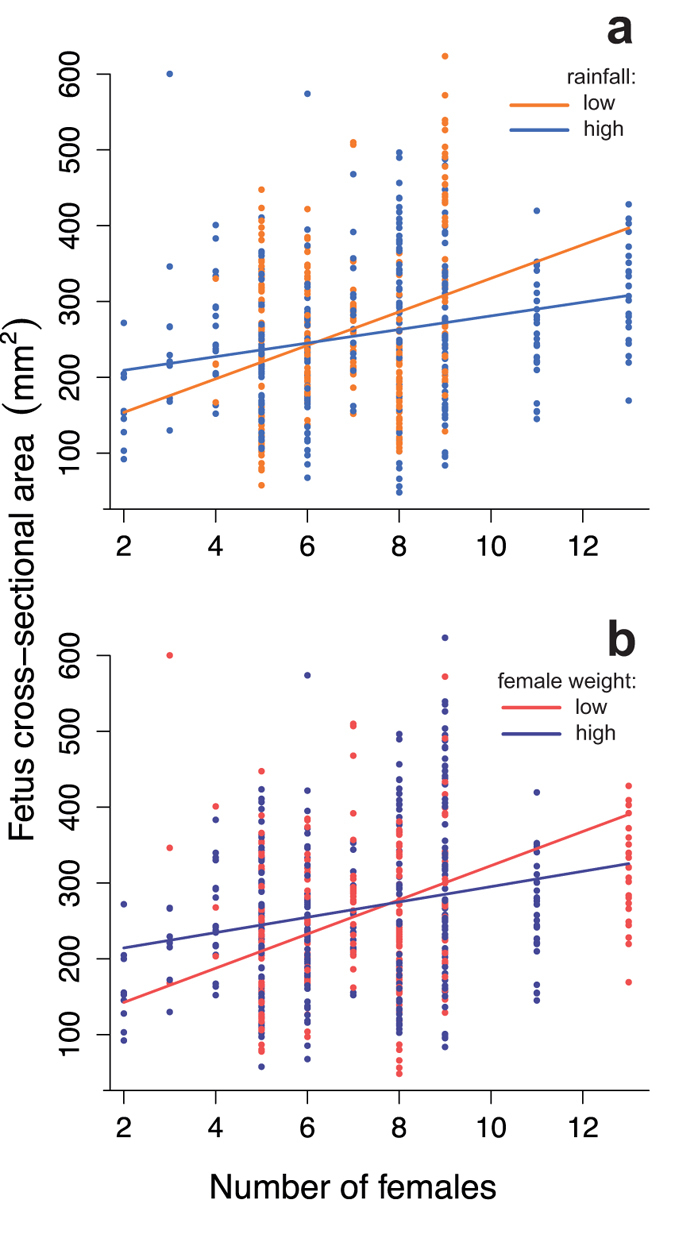
Variation in prenatal investment as a function of the number of adult females in the group at conception. (**a**) Fetus cross-sectional area increases more sharply when rainfall is low (orange line) compared to high (light blue line); (**b**) Lighter females (red line) show the steepest increase in fetus size with female number compared to heavier females (dark blue line). Female weight (mean ± sd = 1447 ± 201 g) and rainfall (mean ± sd = 128.3 ± 40.9 ml) are continuous variables that have been categorized for illustrative purposes using the 25% and 75% quartiles.

The total prenatal investment (fetus size x number of fetuses carried) of females increased with the number of other adult females in the group during pregnancy, and with a female’s weight at conception (LMM, number of females, χ^2^_1_ = 5.65, N = 142, *P* = 0.017, female weight: (LMM, χ^2^_1_ = 12.60, N = 142, *P* < 0.001). This relationship was driven by fetus size rather than number: mean fetus size increased with the number of females in the group; increased more steeply in lighter females, and in breeding attempts featuring lower rainfall (LMM, 2 way interaction of female number with: weight, χ^2^_1_ = 4.23, N = 360 scans, *P* = 0.040; rainfall, χ^2^_1_ = 4.91, N = 360, *P* = 0.027; Fig. 2). Neither total group size nor female age influenced fetus size (see Supplementary Information (SI) Table S1). Within-female variation was a better predictor of fetal size in response to reproductive competition than between-female variation (LMM, within-female variation, χ^2^_1_ = 4.51, N = 360, *P* = 0.034, between-female variation, χ^2^_1_ = 3.38, N = 360, *P* = 0.066; SI Table S2). The number of fetuses was only influenced by female age, peaking at 4 years of age before declining (GLMM poisson, χ^2^_1_ = 10.36, N = 361, *P* = 0.001). There was no significant relationship between fetus size and the number of fetuses (LMM, χ^2^_1_ = 1.03, N = 581, *P* = 0.31). Thus individual females produced larger fetuses, but no fewer of them, when faced with competition from other female breeders.

### (2) What are the consequences of variation in prenatal investment for mothers and offspring?

Female reproductive success (number of assigned pups at emergence) increased with the number of fetuses during gestation, (GLMM poisson, χ^2^_1_ = 5.44, N = 153 females, *P* = 0.02; SI Table S3). However, larger fetuses did not translate into a greater number of assigned pups (GLMM poisson, χ^2^_1_ = 0.76, N = 151 pups, *P* = 0.38). Fetus size also did not influence pup weight at 3 months (LMM, χ^2^_1_ = 0.37, N = 115 pups, *P* = 0.54; SI Table S4), nor survival to 3 months (GLMM, binomial, χ^2^_1_ = 0.12, N = 131 pups, *P* = 0.72). Relative fetus size (measured relative to other scanned females in a particular breeding attempt) also did not predict a female’s share of total group reproductive success (GLMM binomial, χ^2^_1_ = 1.14, N = 153, *P* = 0.29) nor pup survival to 3 months (GLMM binomial, χ^2^_1_ = 1.09, N = 131, *P* = 0.30). Thus, we found no evidence that the production of larger fetuses translated into improved success in postnatal reproductive competition, at least in the short term.

**Figure 3:**
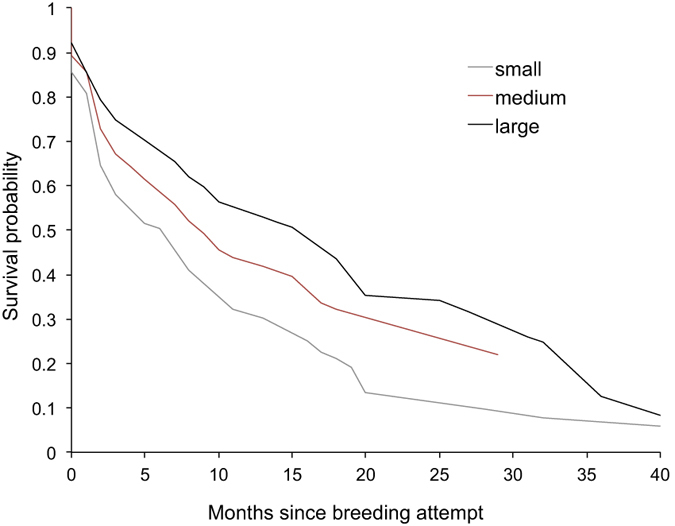
Maternal survival as a function of prenatal investment. Mothers that invested more prenatally survived longer. Fetus size (mean ± sd = 247.90 ± 100.88 mm^2^) has been categorized for illustrative purposes using the 25% (179.54 mm^2^), mean and 75% (319.09 mm^2^) quartiles.

Finally, we found no evidence of a cost of prenatal investment to mothers in terms of future survival or reproduction. In fact, higher total prenatal investment was associated with higher post-scan survival of mothers (Cox regression, Wald χ^2^_1_ = 6.57, N = 360, P = 0.010; Fig. 3). Again this relationship was driven by fetus size rather than number (SI Table S5). Females that invested more prenatally were not less likely to reproduce in the next breeding attempt (GLMM binomial, χ^2^_1_ = 0.35, N = 164, P = 0.061; SI Table S6). Thus we found no evidence of a survival cost to mothers of elevated prenatal investment, nor did mothers compensate for high prenatal investment by reducing reproductive effort in the next breeding attempt.

## Discussion

Female banded mongooses produced larger, but no fewer, offspring when there were more adult females in the group. Since all adult females breed in most breeding attempts, this is consistent with the hypothesis that females strategically up-regulate prenatal investment in the face of elevated postnatal reproductive competition. Such responses may be particularly likely to evolve in breeding systems where females co-breed regularly. Females showed steeper increases in prenatal investment when ecological conditions were harsh, and when they were in relatively poor body condition, two factors which are expected to exacerbate the intensity of postnatal competition among offspring^34^. We found no evidence that increased prenatal investment incurred future costs to females in terms of reproduction or survival. On the contrary, females that invested more prenatally showed improved future survival (Fig. 3). A positive relationship between current reproductive investment and future survival is expected where females vary considerably in quality or access to resources, since high quality females may be able to divert more resources to offspring production without compromising their somatic function (the ‘big house big car’ effect^35^,^36^).

Increasing fetus size in response to increased social competition is a subtle way in which females could compete over reproduction within social groups without risking the costs of fighting or killing offspring^3^,^4^. However, we found no detectable benefit (in terms of short-term reproductive success) associated with increased investment in fetus size. Neither absolute fetus size nor fetus size relative to other co-breeders predicted the number of offspring that survived to emerge from the den. The lack of any detectable advantage to elevated prenatal investment is surprising, and may reflect a high level of noise associated with high pup mortality due to intra- or intergroup infanticide and predation^18^,^19^. It may also be that the benefits of increased prenatal investment are realised later in the life of the offspring. Studies of human famine and laboratory rodents, for example, suggest that early life environments can influence health and fitness across the lifespan, not just in the short term^13^.

Our findings offer an interesting contrast to studies of social birds and fish, in which dominant females produce smaller eggs or a larger number of eggs when there are many helpers in the group^37^,^38^,^39^,^40^. In banded mongooses, all group members contribute to rearing young, but prenatal investment did not vary with the potential number of helpers (measured by total group size). Our findings suggest that the intensity of reproductive competition, rather than the availability of helpers, is the main determinant of variation in prenatal investment in this species. Larger pups have better access to adult group members who provide parental care and, upon emergence, aggressively defend access to the best helpers or ‘escorts’^41^. Where postnatal competition among offspring has characteristics of contest competition, the best response to competition will be to invest more resources per offspring prenatally, rather than to produce more of them^42^,^43^. Producing a larger number of offspring could also bring benefits, but at the unavoidable cost of intensified competition among littermates.

Our study complements previous studies which suggest that mothers use hormones to influence the development of their offspring *in utero* to improve their success in the postnatal environment, a form of PAR^13^,^44^. The PAR hypothesis has been criticized because long term forecasts of environmental conditions are inherently unreliable^14^,^15^. In cooperative breeders, however, the quality of the postnatal environment is largely determined by the number of breeders competing for reproduction and the number of helpers available to offspring. These features of social groups remain stable over the course of offspring development, from gestation to nutritional independence, so are highly predictable. Cooperative birds and mammals, including humans, are thus likely candidates to evolve PARs. We found evidence that female banded mongooses respond to reproductive competition by adjusting prenatal investment, consistent with the PAR hypothesis, but we did not find evidence that this response is adaptive. To test the PAR hypothesis fully will require study of the consequences of variation in prenatal investment across the lifetime of offspring in animals exposed to natural predators and pathogens.

## Additional Information

**How to cite this article**: Inzani, E. L. *et al.* Female reproductive competition explains variation in prenatal investment in wild banded mongooses. *Sci. Rep.* **6**, 20013; doi: 10.1038/srep20013 (2016).

## Supplementary Material

Supplementary Tables
