# Supplementary material for: Female reproductive competition explains variation in prenatal investment in wild banded mongooses
Source: Sci Rep. 2016 Jan 28;6:20013. doi: 10.1038/srep20013 (PMC4730225; doi:10.1038/srep20013)
Supplement: Supplementary Tables [file srep20013-s1.doc]

**Female reproductive competition explains variation in prenatal investment in wild banded mongooses**

Emma L. Inzani, Harry H. Marshall, Sarah J. Hodge, Jennifer L. Sanderson, Hazel J. Nichols, Faye J. Thompson, , Gladys Kalema-Zikusoka, Michael A. Cant* & Emma I.K. Vitikainen

Centre for Ecology and Conservation, University of Exeter, Penryn Campus, Cornwall TR10 8FE

*Corresponding author: m.a.cant@exeter.ac.uk

| **Supplementary Information**  **Table S1: Factors affecting prenatal investment** | | | | | | | | | | | | | | | |
| --- | --- | --- | --- | --- | --- | --- | --- | --- | --- | --- | --- | --- | --- | --- | --- |
|  | **Fetus cross-sectional area (fetus size)** | | | | | **Number of fetuses** | | | | | **Total prenatal investment**  **(fetus size x number of fetuses)** | | | | |
| **Model terms** | **Effect size ± SE** | | | **χ^2^** | ***P*** | **Effect size ± SE** | | | **χ^2^** | ***P*** | **Effect size ± SE** | | | **χ^2^** | ***P*** |
| **Female age (months)** | -0.12 | ± | 0.38 | 0.10 | 0.75 | **0.12** | **±** | **0.038** | **10.36** | **0.0013** | 1.8 | **±** | 1.5 | 1.34 | 0.25 |
| **Female age^2^ (months)** | -0.0018 | ± | 0.0042 | 0.087 | 0.77 | **-0.053** | ± | **0.025** | **4.81** | **0.028** | 0.021 | ± | 0.016 | 1.56 | 0.21 |
| **Female weight (g)** | 0.30 | ± | 0.10 |  |  | 0.036 | ± | 0.033 | 1.19 | 0.27 | **0.58** | **±** | **0.16** | **12.60** | **<0.001** |
| Rainfall during pregnancy (ml) | 1.81 | ± | 0.70 |  |  | -0.0079 | ± | 0.031 | 0.068 | 0.80 | -0.68 | **±** | 0.85 | 0.64 | 0.42 |
| **Number of females** | -53 | ± | 57 |  |  | -0.0094 | ± | 0.031 | 0.09 | 0.76 | **38** | **±** | **15** | **5.65** | **0.017** |
| **Female weight x number of females** | **-0.029** | **±** | **0.014** | **4.23** | **0.040** |  |  |  |  |  |  |  |  |  |  |
| **Total rainfall x number of females** | **-0.24** | **±** | **0.11** | **4.91** | **0.027** |  |  |  |  |  |  |  |  |  |  |
| Group size | 1.8 | ± | 2.6 | 0.089 | 0.77 | 0.0012 | ± | 0.03 | 0.0015 | 0.97 | -9.9 | **±** | 10 | 0.85 | 0.36 |
| Fetus age (days) | -45 | ± | 20 |  |  |  |  |  |  |  |  |  |  |  |  |
| Fetus age^2^ (days) | 0.64 | ± | 0.29 |  |  |  |  |  |  |  |  |  |  |  |  |
| Sample | 360 ultrasounds from 59 females in 41 litters from 8 groups. | | | | | 361 observations from 127 females in 130 litters from 11 groups | | | | | 360 ultrasounds from 59 females in 41 litters from 8 groups. | | | | |
| Random effects: female ID, litter ID and group ID. Model terms were scaled in GLMM analysis on number of fetuses. | | | | | | | | | | | | | | | |

| **Table S3: Consequences of prenatal investment – female reproductive success** | | | | | | | | | | |
| --- | --- | --- | --- | --- | --- | --- | --- | --- | --- | --- |
|  | **Number of emergent pups assigned to female** | | | | | **Proportion of pups in a group litter assigned to female** | | | | |
| **Model terms** | **Effect size ± SE** | | | **χ^2^** | ***P*** | **Effect size ± SE** | | | **χ^2^** | ***P*** |
| Mean fetus size (mm^2^) | 0.0022 | ± | 0.0017 | 1.66 | 0.20 |  |  |  |  |  |
| Relative fetus size |  |  |  |  |  | 0.0023 | ± | 0.0022 | 1.14 | 0.29 |
| Number of fetuses | 0.28 | **±** | 0.12 | 5.44 | 0.020 |  |  |  |  |  |
| Fetus age (days) | 0.05 | ± | 0.03 |  |  | 0.0025 | ± | 0.0017 |  |  |
| Sample | 153 observations from 78 females in 51 litters from 10 groups. | | | | | 153 observations from 78 females in 51 litters from 10 groups. | | | | |
| Random effects: female ID, litter ID and group ID. | | | | | | | | | | |

| **Table S2: Within- and between- female variation in fetus size** | | | | | |
| --- | --- | --- | --- | --- | --- |
| **Model terms** | **Effect size** | **±** | **SE** | **χ^2^** | ***P*** |
| Female age (months) | 0.78 | ± | 0.35 | 0.045 | 0.83 |
| Female age^2^ (months) | 0.00061 | ± | 0.0041 | 0.021 | 0.89 |
| Female weight at conception (g) | 0.064 | ± | 0.043 | 2.00 | 0.16 |
| Rainfall during pregnancy (ml) | -0.094 | ± | 0.23 | 0.17 | 0.68 |
| **Within-female effects** | **12.24** | ± | **5.63** | **4.51** | **0.034** |
| Between-female effects | 9.55 | ± | 4.76 | 3.38 | 0.066 |
| Fetus age (days) | 2.88 | ± | 1.20 |  |  |
| Fetus age^2^ (days) | -0.048 | ± | 0.093 |  |  |
| Sample | 360 ultrasounds from 59 females in 41 litters from 8 groups. | | | | |
| Random effects: female ID, litter ID and group ID. | | | | | |

| **Table S4: Consequences of prenatal investment – Pup survival and growth to independence** | | | | | | | | | | |
| --- | --- | --- | --- | --- | --- | --- | --- | --- | --- | --- |
|  | **Pup survival to 3 months** | | | | | **Pup growth (age<=90 days)** | | | | |
| **Model terms** | **Effect size ± SE** | | | **χ^2^** | ***P*** | **Effect size ± SE** | | | **χ^2^** | ***P*** |
| Mean fetus size (mm^2^) | 0.0014 | ± | 0.0041 | 0.12 | 0.72 | 0.04 | **±** | 0.06 | 0.34 | 0.56 |
| Relative fetus size (mm^2^) | 0.0063 | ± | 0.0023 | 1.09 | 0.30 |  |  |  |  |  |
| Number of fetuses | 0.02 | ± | 0.27 | 0.0058 | 0.94 | -7.0 | **±** | 5.5 | 1.59 | 0.21 |
| Total number of pups in a group litter | -0.05 | **±** | 0.10 | 0.23 | 0.63 | -0.94 | **±** | 1.55 | 0.37 | 0.54 |
| Sex of pup |  |  |  |  |  | 9.68 | **±** | 10.30 | 0.87 | 0.35 |
| Pup age (days) |  |  |  |  |  | 4.0 | **±** | 1.1 |  |  |
| Fetus age (days) | 0.02 | ± | 0.06 |  |  | 4.1 | **±** | 1.0 |  |  |
| Sample | 131 pups from 29 litters from 8 groups. | | | | | 116 pups from 26 litters from 8 groups. | | | | |
| Random effects: litter ID and group ID. | | | | | | | | | | |

| **Table S5: Consequences of prenatal investment– female survival**  Female post-reproductive survival (months). Cox regression with backward selection of terms (Wald). | | | | | |
| --- | --- | --- | --- | --- | --- |
| **Model terms** | **Effect size** | **±** | **SE** | **Wald χ^2^** | ***P*** |
| Number of females | - 0.081 | **±** | 0.0052 | 2.23 | 0.14 |
| **Total prenatal investment** | **-0.001** | **±** | **0.0001** | **6.57** | **0.010** |
| Number of fetuses | -0.009 | **±** | 0.121 | 0.006 | 0.94 |
| **Mean fetus size (mm^2^)** | **-0.005** | **±** | **0.001** | **12.68** | **<0.001** |
| Relative fetus size (mm^2^) | 0.001 | **±** | 0.003 | 0.16 | 0.69 |
| Sample | 109 females in 47 litters from 10 groups. | | | | |

| **Table S6: Consequences of prenatal investment – female participation in next litter (y/n)** | | | | | |
| --- | --- | --- | --- | --- | --- |
| **Model terms** | **Effect size** | **±** | **SE** | **χ^2^** | ***P*** |
| Female age (months) | -0.01 | ± | 0.02 | 0.17 | 0.68 |
| Female age^2^ (months) | -0.0015 | ± | 0.0029 | 0.28 | 0.60 |
| Female weight at conception (g) | 0 | **±** | 0.0031 | 0.012 | 0.91 |
| Number of females | 0.19 | **±** | 0.48 | 0.16 | 0.69 |
| Mean fetus size (mm^2^) | -0.0012 | **±** | 0.0072 | 0.030 | 0.86 |
| Number of fetuses | -0.87 | **±** | 0.52 | 3.50 | 0.061 |
| Fetus age (days) | 0.06 | **±** | 0.14 |  |  |
| Sample | 105 observations from 46 females in 34 litters from 7 groups. | | | | |
| Random effects: female ID, litter ID and group ID. | | | | | |
